# Needlescopic sutureless repair of congenital inguinal hernia: A randomized controlled study

**DOI:** 10.1007/s13304-023-01566-9

**Published:** 2023-06-21

**Authors:** Rafik Shalaby, Mohamed Abdelmaboud, Mohammad Daboos, Yousef Mohamed, Ahmed Abdelghafar Helal, Ibrahim Gamman

**Affiliations:** Pediatric Surgery Departments, Faculty of Medicine, Al-Azhar University, Cairo, Egypt

**Keywords:** Needlescopic, Separation, Mediflex, Diathermy probe, Infant and children, Epidural needle

## Abstract

Congenital inguinal hernia [CIH] can be treated laparoscopically using various methods documented in the literature. Many authors have recommended dividing the sac and stitching peritoneal defects. Other studies claimed that peritoneal disconnection alone is sufficient. In this study, the feasibility, operative time, recurrence rate, and other postoperative complications of needlescopic disconnection of the CIH sac with or without peritoneal defect suturing were compared. A prospective controlled randomized trial was conducted between January 2020 and December 2022. Two hundred and thirty patients who met the study requirements were included. Patients were assigned at random to either Group A or Group B. A group of 116 patients (Group A) had needlescopic separation of the neck of the sac and peritoneal defect closure. The remaining 114 patients (Group B) underwent needlescopic separation without peritoneal defect closure (Sutureless group). A total of 260 hernial defects in 230 patients were repaired using needlescopic disconnection with or without suturing of the defect. There were 89 females (38.7%) and 141 males (61.3%), with a mean age of 5.14 ± 2.79 years. In Group A, the mean operation time was 27.98 ± 2.89 for a unilateral hernia and 37.29 ± 4.68 for a bilateral one, whereas, in Group B, the mean operation time was 20.37 ± 2.37 and 23.38 ± 2.22 for a unilateral and bilateral hernia, respectively. In terms of the operating time, whether unilateral or bilateral, there was a significant difference between the groups. There was no significant difference between groups A and B in the mean Internal Ring Diameter [IRD], which was 1.21 ± 0.18 cm in group A and 1.19 ± 0.11 cm in group B. Throughout the follow-up period, there was no postoperative hydrocele formation, recurrence, iatrogenic ascending of the testes, or testicular atrophy. All patients had nearly invisible scars with no keloid development at 3 months follow-up. Needlescopically separating the hernia sac without stitching the peritoneal defect is feasible, safe, and less invasive. It provides outstanding cosmetic results with a short operative time and no recurrence.

## Introduction

Congenital inguinal hernia [CIH] is a common surgical issue seen by pediatric surgeons. It accounts for approximately 15% of all pediatric surgical procedures [1]. For many decades, open herniotomy has been the standard treatment for CIH by many pediatric surgeons [2, 3]. Laparoscopy, however, has gained acceptance for CIH repair because of recent advances in minimally invasive surgery [4, 5]. Many options are available for laparoscopic inguinal hernia repairs in children [6, 7]. Laparoscopic separation of the hernia sac with peritoneal suturing around the Internal Inguinal Ring [IIR] was used to imitate an open herniotomy and to reduce recurrence [8]. However, other studies have found that separating the sac at its neck without peritoneal suturing is a good treatment, particularly in hernias with small internal ring diameters (IRD) [6, 8]. Others have determined that suturing is recommended for larger rings up to 20 mm in diameter. Furthermore, some authors have claimed that separating the peritoneal sac alone, or even partial cauterization of the neck, is effective in wide rings up to 2 cm in diameter, or even regardless of their dimensions [9, 10]. However, to the best of our knowledge, no studies have compared separation with peritoneal suturing to separation without peritoneal suturing using needlescopic instruments. This study aimed to compare the feasibility, operative time, recurrence rate, and cosmetic results of needlescopic separation of the hernial sac with peritoneal closure and separation alone.

## Patients and methods

This prospective controlled randomized trial was conducted et al.-Azhar University Hospital’s, Department of Pediatric Surgery in Cairo, Egypt, between January 2020 and December 2022. The study design was approved by our medical school's Institutional Review Board [IRB] No. 0000395, and informed consent was obtained from the patient’s parents. Patients were randomly assigned to either Group A or Group B. Patients in Group A underwent needlescopic separation of the neck of the sac together with peritoneal defect closure, whereas patients in Group B underwent needlescopic separation alone without peritoneal defect closure. Children of both sexes with CIH who underwent CIH repair by needlescopic separation of the neck of the sac with or without peritoneal defect suturing over the IIR were eligible for inclusion. Patients who underwent different forms of hernia repair, including needlescopy with purse-string IIR without peritoneal division or muscular arch repair, were excluded from the study. The operation time was the primary outcome of this study. The secondary outcomes were recurrence, testicular atrophy, hydrocele development, and iatrogenic testicular ascent.

### Instruments

1. A 5-mm trocar for a 30° telescope, 2. A Suture Grasper Device [SGD], 3. An 18-G epidural needle [EN], 4. A long isolated homemade diathermy probe [DP]connected to an ordinary diathermy handle. The DP was made by thinning out a Kirschner's wire to 1.5 mm and isolating it with a shrinkable rubber tube.

### Sites of port and needles

**Fig. 1:**
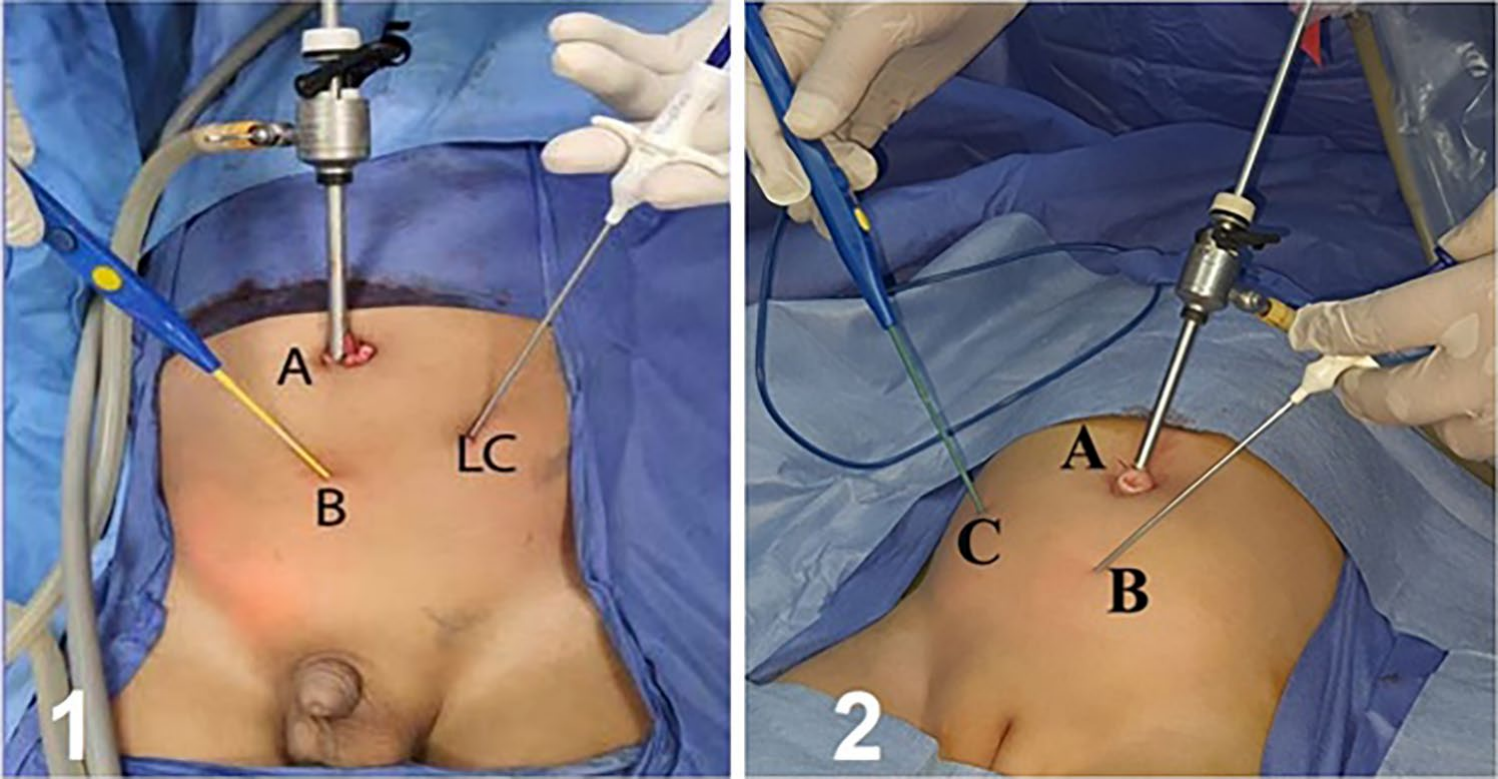
Sites of camera port and needles for **(1)** left-sided CIH and **(2)** right-sided CIH. [1-**A**, Camera port. 1-**B**, site of insertion of DP. 1-**LC**, site of insertion of SGD. 2-**A**, Camera port, 2-**B**, site of insertion of SGD. 2-**C**, site of insertion of DP]

Point A: vertical umbilical incision within the umbilical cicatrix for a 5-mm port. Point B: a tiny stab puncture in the midline [midway between the umbilicus and pubis]. Point C: a tiny stab puncture at the corresponding McBurney’s point (point RC in right-sided hernia and point LC in left-sided ones) as shown in (Fig. 1).

### Operative details

We used the approach outlined by Shalaby et al. [11]. For further information, the reader is directed to Shalaby et al. [11], Marey et al. [12], and supplementary digital content: http://links.lww.com/SLE/A300https://drive.google.com/file/d/1sj-9vrdojACSLq7rEGq7EUI5XLpmsNYz/view?usp=sharing).

**Fig. 2:**
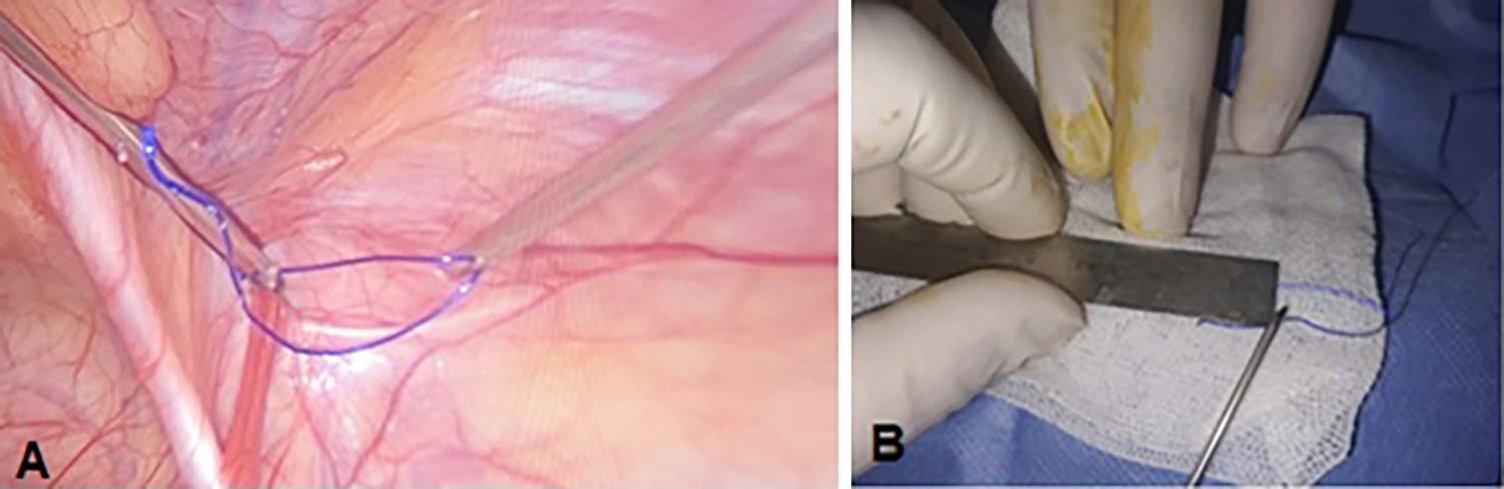
Laparoscopic view of the right CIH in a male child. **A**, A piece of polypropylene suture was grasped between two SGDs to measure the widest diameter of the IIR. **B**, the suture was then taken outside, and the length was measured using a regular ruler graded by mm

**Fig. 3:**
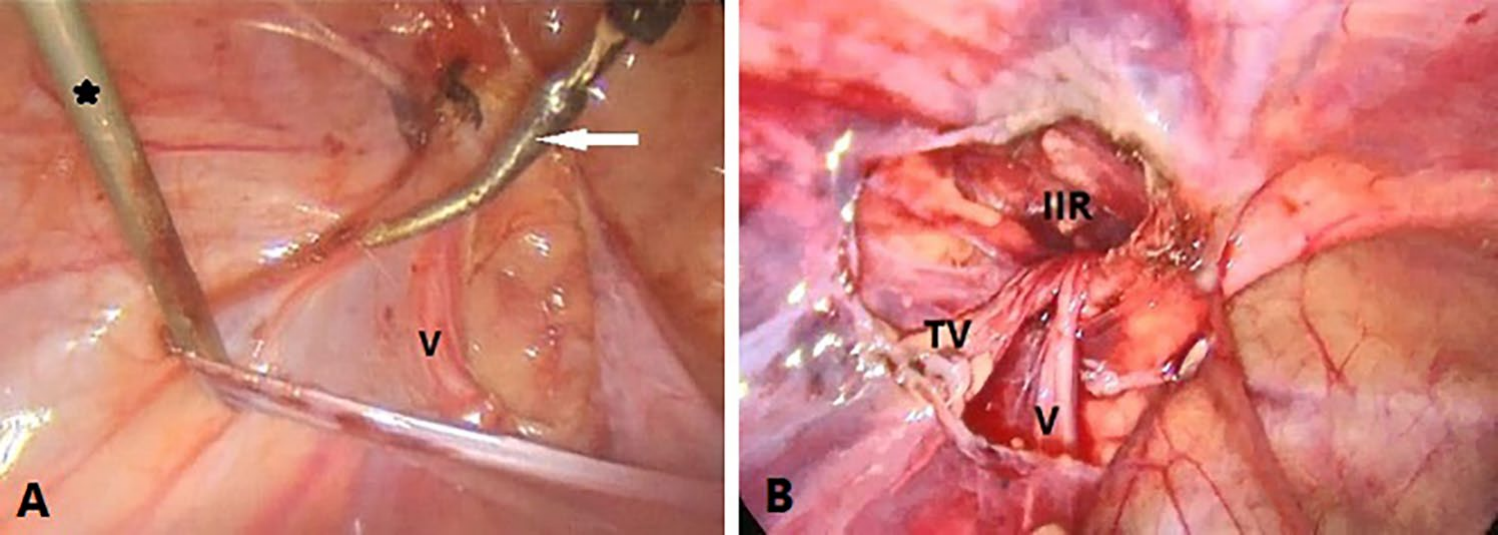
Steps of separating the hernia sac for left-sided CIH in a male child. **A** The vas and testicular vessels were swept off by blunt dissection using DP. **B** Complete hernial sac separation. *V*  vas deferance, *TV* testicular vessels, *IIR* internal inguinal ring, black asterisk SGD, white arrow  DP

*In Both Groups:* The IRD was measured using two SGD and a piece of polypropylene suture (2/0) 10 cm in length (Fig. 2). The peritoneum was then grabbed and pulled away from the vas and testicular blood vessels by using SGD at the neck of the sac. The hernia sac was then carefully separated using DP. The testicular blood vessels and vas deference were carefully swept off by blunt dissection (Fig. 3).

**Fig. 4:**
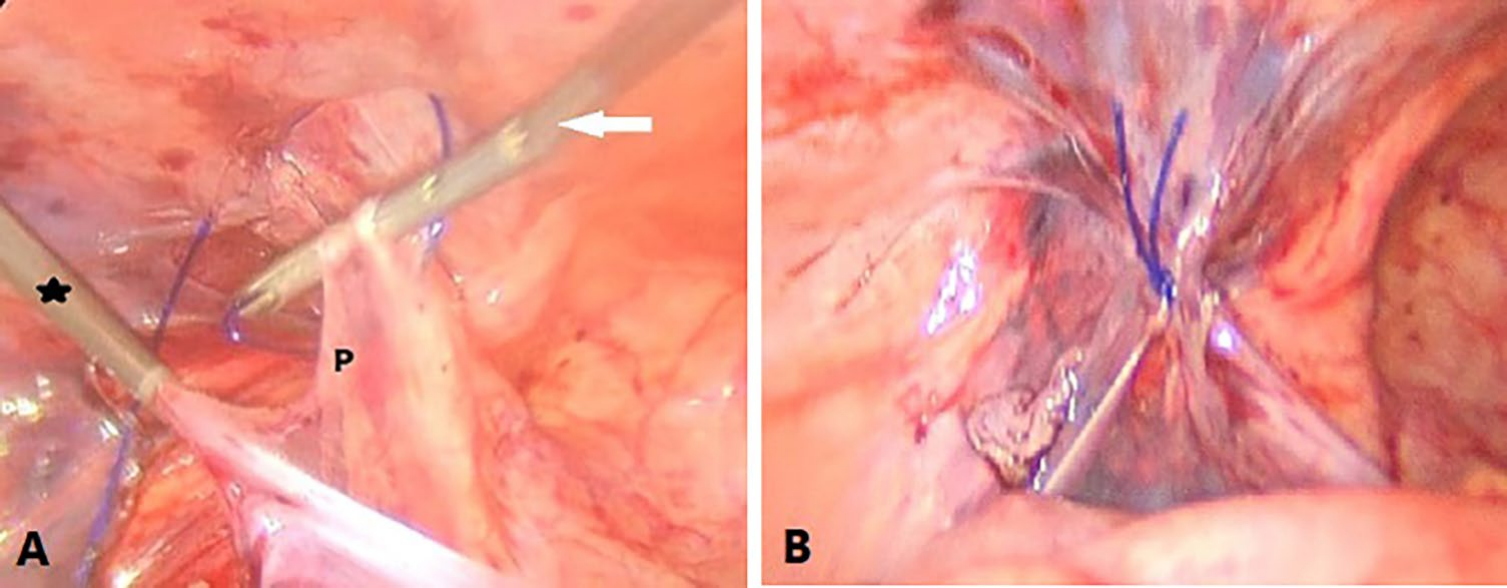
Steps of closing the peritoneal defect at IIR for left-sided CIH in a male child. **A** EN threading the Peirce string suture. **B** photo was taken after ligation of the suture and cutting both ends outside the abdomen. *P* peritoneum, Black asterisk SGD, white arrow EN

*In Group A,* a pierce-string suture via EN was used to seal the peritoneal defect (Fig. 4). Subsequently, both suture ends were grabbed and extracted from the same point using SGD. A French sliding knot was formed, advanced, and then moved back. The ends of the sutures were grabbed and dragged outward. Following deflation of the abdomen, they were cut flush with regular scissors outside the abdomen.

*In Group B,* the same steps were repeated without suturing the peritoneal defect.

The port and instruments were removed. A 3/0 polyglactin suture (Vicryl) was used to close the umbilical incision. Only Steri-Strips were used for the other incisions.

The concerned physicians conducted a follow-up during routine outpatient visits to search for postoperative complications. Cosmetic success was assessed based on the satisfaction of the parents with the appearance of the scar. Parental satisfaction was measured using a subjective score ranging from 0 to 4, where 0 represented poor, 1 represented fair, 2 represented good, 3 represented very good, and 4 represented excellent.

### Statistics

The Statistical Package for Social Science (SPSS, version 23) was used to analyze the collected data. Parametric data are presented as means and standard deviations. Numbers and percentages were used to represent the qualitative characteristics. The Kolmogorov–Smirnov test was used to assess the normality of the data distribution. Using qualitative data, the chi-square test was used to compare the groups. The comparison between two groups with quantitative data and parametric distribution was done by using an independent *t* test.

## Results

A total of 230 patients with 260 hernial defects were treated by needlescopic division with or without suturing of the peritoneal defect. They comprised 141 males (61.3%) and 89 females (38.7%), with a mean age of 5.14 ± 2.79 years. The patients were randomized into groups A (*N* = 116) and B (*N* = 114). There were no significant differences in the preoperative patient characteristics or clinical data between the two groups (Table 1).

**Table 1:** Showing comparison between groups as regard demographic data according to the number of patients

Preoperative parameters	Group A No. = 116	Group B No. = 114	*P* value
Age/year (Mean ± SD)	5.29 ± 2.85	4.62 ± 2.14	0.168
Sex			
Female	45 (38.8%)	44 (57.5%)	0.089
Male	71 (61.2.8%)	70 (42.5%)	
Side of hernia			
Right	51 (39.8%)	60 (52.6%)	0.491
Left	49 (38.3%)	40 (35.0%)	
Bilateral	16 (16.0%)	14(12.8.0%)	

*P* value < 0.05 is Significant

Initial diagnostic laparoscopy revealed 200 patients with unilateral inguinal hernia, 100 in group A and 100 in group B. Thirty patients had bilateral hernias, 16 in group A and 14 in group B. Other demographic data of all patients are shown in (Table 1).

In Group A, the mean operation time was 27.98 ± 2.89 for a unilateral hernia and 37.29 ± 4.68 for a bilateral one, whereas, in Group B, the mean operation time was 20.37 ± 2.37 and 23.38 ± 2.22 for a unilateral and bilateral hernia, respectively. There was a significant difference between the groups in terms of operating time, whether unilateral or bilateral (Table 2). There was no significant difference between groups A and B in the mean (IRD), which was 1.21 ± 0.18 cm in group A and 1.19 ± 0.11 cm in group B. No intraoperative difficulties were observed in either study group, except for one patient in group B who experienced bleeding due to inferior epigastric blood vessel injury. However, bleeding was stopped by deflating the abdomen and compressing it for 5 min. All surgeries were completed without conversion to conventional laparoscopy or open repairs. Most patients [97.4% in group A and 96.5% in group B] were discharged on the same day of operation.

**Table 2:** Showing comparison between groups as regards operative time, and intraoperative complications according to the number of hernias

Operative parameters	Group A (No. = 132)	Group B (No. = 128)	*P* value
Operative time (Mean ± SD)			
Unilateral hernia	27.98 ± 2.89	20.37 ± 2.37	< 0.001
Bilateral hernia	37.29 ± 4.68	23.38 ± 2.22	< 0.001
Size of IIR/cm	1.21 ± 0.18	1.19 ± 0.11	0.441
Bleeding			
No	132 (100.0%)	128 (98.18%)	0.544
Yes	0 (0.0%)	1 (1.82%)	

*P* value < 0.05 is Significant. Asterisk = significant *p* value

For a mean of 1.5 ± 0.5 years, all patients were followed up on routine outpatient visits. The concerned physicians evaluated the patients postoperatively after one week, two weeks, one month, three months, and one year to a maximum of two years for the presence of complications. No postoperative hydrocele, recurrence, testicular ascent, or atrophy was observed. However, there were four cases of postoperative umbilical infections in group A and five cases in group B. With watchful treatment, these infections improved significantly (Table 3).

**Table 3:** Showing comparison between groups as regards postoperative complications and cosmetic outcomes according to the number of patients and time of follow-up

Postoperative parameters			Group A (No. = 116)		Group B %(No. = 114) %		*P* value
Infection	1 week	No	112	96.5	109	95.6	0.577
		Yes	4	3.4	5	4.4	
	2 weeks-2 years	No	116	100	114	100	NA
		Yes	0	0	0	0	
Cosmetic outcome	1 week—2 years	Good	6	5.2	0	0.0	0.116
		Very good	12	10.3	11	9.6	
		Excellent	98	84.5	103	90.4	

*P* value < 0.05 is Significant

In the context of aesthetic results, Group A included 98 patients (84.5%) with excellent cosmoses, 12 (10.3%) with very good cosmoses, and 6 (5.2%) with good cosmoses after the first week. In Group B, 103 patients (90.4%) had excellent cosmoses, whereas 11 (9.6%) had very good cosmoses. There were no statistically significant differences between the two groups during any of the follow-up periods (Table 3).

## Discussion

Congenital inguinal hernia is one of the most common surgical procedures performed in infants and children. For several decades, open herniotomy with transection-ligation of the hernial sac has been considered the gold standard treatment approach [1]. According to proponents of open repair, laparoscopy has a higher recurrence rate [13]. The reported recurrence rates for laparoscopic hernia correction, which varied between 0.3 and 1.2%, reached or even dropped below those of open surgery, with more experience and optimum procedure selection [13–15]. The benefits of laparoscopic inguinal hernia repair include excellent visual exposure; the ability to detect the contralateral hernia; minimum dissection; avoidance of trauma to the vas deferens, testicular blood vessels, and other adjacent structures; reduced wound infection rate; less discomfort; and shorter hospital stay [2]. Many meta-analyses have shown no significant difference between laparoscopy and open herniotomy in terms of operative time and recurrence rates [4–6].

Compared with open surgery, Esposito et al. [16] found that laparoscopic peritoneal separation and suturing of the proper neck had the lowest recurrence rate. The fundamental reason for the low recurrence rate is believed to be peritoneal injury during dissection, followed by scarring [17, 18]. Compared to open herniotomy, needlescopic repair of congenital inguinal hernias is safe and feasible. Furthermore, the outstanding aesthetic outcomes gained from needlescopic surgery encouraged some surgeons to employ this technique [19, 20]. Therefore, we aimed to benefit from the positive effects of needlescopic surgery and hernial sac disconnection.

Regarding the operative time, we found a significant difference between the study groups for unilateral or bilateral hernias. According to Shalaby et al. [11], the average operative time for a unilateral hernia was 14.28 ± 2.98 min and 23.36 ± 4.67 min for a bilateral one.

During a mean follow-up period of 1.5 ± 0.5 years, we detected no evidence of delayed postoperative complications or recurrence. The 0% recurrence rate in both groups in this study can be explained by the fact that all surgeries were performed by the same group of surgeons. This result was aided by careful and liberal peritoneal disconnections. In agreement with our results, many authors have reported a 0–2.9% recurrence rate using the sac division and suturing technique [1–18]. With sac separation, Riquelme et al. [21] and Prasad et al. [22] reported a 0% recurrence rate; however, Riquelme et al. [21] closed internal rings larger than 10 mm. Compared with no recurrence (0%) in the separation and suturing group, Elbatarny et al. [23] found a worrying recurrence of hernia (20%) in 3 15 patients in the separation-only group. However, they reported recurrence in patients with IRDs greater than 10 mm. With no statistically significant difference, Pant et al. [10] reported that recurrence occurred in one of 34 hernias (2.9%) in the separation group and in two of 38 hernias (5.3%) in the separation and suturing groups. According to Garca-Hernández et al. [24], employing the separation-only approach, recurrence occurred in two cases (0.53%), regardless of the IRD. However, the sac was completely removed. Using separation alone, Shehata et al. [25] reported 0% recurrence in 20.5 months of follow-up; however, they closed the internal rings > 20 mm.

We decided to use 1.5 cm as the upper limit for the internal ring size. Above this point, peritoneal closure with narrowing of the IIR was advised, as in Shehata et al. [14]. They classified hernia as Pediatric Nyhus (PN); PNI, PNII, and PNIII, with mean IRD of 7.7 mm ± 1.5, 16.7 mm ± 3.6, and 22.6 mm ± 4.6, respectively. The PNI was assigned for herniotomy alone, PNII for herniotomy plus IIR narrowing, and PNIII for herniotomy plus posterior wall repair. Following prior recommendations, a 0% recurrence rate was recorded in all the cases.

Hydroceles following laparoscopy are related to a variety of variables [12]. However, the most significant one may be the non-division of the sac. [17, 18] On 33 cases with bilateral CIH, Almetaher et al. [15] used laparoscopic purse-string suturing on one side and laparoscopic division and suturing at IIR on the other. They concluded that separation of the sac with peritoneal closure was significantly superior. Takehara et al. [13] reported that ligating the IIR alone without dividing the sac resulted in a high recurrence rate and hydrocele formation.

In contrast to the inguinal skin crease incision, which is mostly hidden and almost invisible after open repair, scars from the working ports may be obvious after conventional laparoscopy. In this study, we replaced working ports with 1.6 mm needle punctures, which were virtually undetectable after 3 months. In terms of cosmetic evaluation, we agree with the findings of Marey et al. [12]. They used parents' subjective satisfaction with the appearance of scars. It was excellent in 31 (93.93%) cases and very good in only 2 cases, with a slight alteration in the shape of the umbilicus. We know that our technique for cosmetic evaluation is entirely subjective, but the presence of nearly invisible scars proves the assessment beyond doubt.

### Study limitations

This study had some limitations, as it was based on a small number of cases. There was a selection bias due to the exclusion of patients with IIR > 15 mm. To ensure safety and raise the level of evidence for separation without peritoneal closure, a larger number of patients from multiple centers with long-term follow-up is essential.

## Conclusion

Needlescopic separation of the hernial sac without stitching the peritoneal defect is feasible, safe, and minimally invasive. It provides outstanding cosmetic results with a short operative time and no recurrence. We believe that the needlescopic method has a great chance of success as a common substitute for conventional laparoscopic hernia surgery in children. Additional randomized controlled studies with larger sample sizes are required to confirm our findings.

## Data Availability

The datasets used and/or analyzed during the current study are available from the corresponding author but could not be sent owing to the medicolegal aspect of the hospital policy.
